# P-594. Temperature Stability of BPL-1357, a BPL-Inactivated, Whole-Virus, Universal Influenza Vaccine

**DOI:** 10.1093/ofid/ofae631.792

**Published:** 2025-01-29

**Authors:** Camellia Banerjee, Luca Tudor Giurgea, Adriana Cervantes-Medina, Jeffery Taubenberger, Matthew J Memoli

**Affiliations:** National Institutes of Health, Bethesda, Maryland; National Institute of Allergy and Infectious Diseases, Bethesda, MD; National Institute of Allergy and Infectious Diseases, Bethesda, MD; National Institute of Allergy and Infectious Diseases, Bethesda, MD; National Institute of Allergy and Infectious Diseases, Bethesda, MD

## Abstract

**Background:**

Influenza A viruses (IAV) present a constant public health threat from seasonal epidemics to potential pandemics. Currently approved vaccines perform poorly against mismatched seasonal influenza strains and are unlikely to provide any protection against a pandemic potential influenza such as avian influenza. We have developed a vaccine composed of four β-propiolactone-inactivated (BPL-inactivated) low-pathogenicity avian IAV subtypes (H1N9, H3N8, H5N1 and H7N3) which demonstrated protection against lethal challenge with a wide variety of influenza subtypes in mice and ferrets. Human trials are ongoing but even with a promising vaccine, thermal stability can still be an issue that could limit use in resource-poor settings.

**Methods:**

The four components of BPL-inactivated vaccine were stored individually at four different conditions, -80°C, -20°C, 4°C, and room temperature. Hemagglutination (HA), neuraminidase activity (NA), and protein assays were done for each temperature condition after 7 and 28 days of storage.

**Results:**

Baseline protein concentrations were similar across vaccine components. After 28 day incubation, protein concentrations remained stable at room temperature and at 4°C for H1N9 and H7N3, but decreased for H3N8 and H5N1, more at room temperature to 52.1% and 56.1% of Day 0 concentration respectively. At 4°C there was less loss of protein for those components. Activity of NA and HA varied at baseline; for example H3 and H5 had much higher activity (1280) than H1 (160) and H7 (320) and NA activity was highest for N1 (482.22 mU/ml) and lowest for N9 (33.65 mU/ml). HA and NA activity was stable for 28 days at room temperature and 4°C. At -20°C, HA activity showed marked declines within 7 days. Overall, our data shows stability of protein and HA and NA activity at 4°C for up to 28 days, and some short-term stability at room temperature.

**Conclusion:**

Developing a universal flu vaccine would contribute greatly to preventing future pandemics. However, for global use, this vaccine would need to be stable and distributable. Here we show that we may potentially have a vaccine that is both efficacious and shelf stable at 4°C. Further evaluation of immunogenicity at different temperature conditions should be performed, especially to resolve discordance between protein and activity assays.

**Disclosures:**

**All Authors**: No reported disclosures

